# A Resampling Method to Improve the Prognostic Model of End-Stage Kidney Disease: A Better Strategy for Imbalanced Data

**DOI:** 10.3389/fmed.2022.730748

**Published:** 2022-03-07

**Authors:** Xi Shi, Tingyu Qu, Gijs Van Pottelbergh, Marjan van den Akker, Bart De Moor

**Affiliations:** ^1^Department of Electrical Engineering (ESAT), Stadius Center for Dynamical Systems, Signal Processing and Data Analytics, KU Leuven, Leuven, Belgium; ^2^Vlerick Business School, Leuven, Belgium; ^3^Department of Computer Science, KU Leuven, Leuven, Belgium; ^4^Department of Public Health and Primary Care, Academic Centre of General Practice, KU Leuven, Leuven, Belgium; ^5^Institute of General Practice, Goethe University, Frankfurt am Main, Germany

**Keywords:** logistic regression, machine learning, resampling method, predictive performance, chronic disease

## Abstract

**Background:**

Prognostic models can help to identify patients at risk for end-stage kidney disease (ESKD) at an earlier stage to provide preventive medical interventions. Previous studies mostly applied the Cox proportional hazards model. The aim of this study is to present a resampling method, which can deal with imbalanced data structure for the prognostic model and help to improve predictive performance.

**Methods:**

The electronic health records of patients with chronic kidney disease (CKD) older than 50 years during 2005–2015 collected from primary care in Belgium were used (*n* = 11,645). Both the Cox proportional hazards model and the logistic regression analysis were applied as reference model. Then, the resampling method, the Synthetic Minority Over-Sampling Technique-Edited Nearest Neighbor (SMOTE-ENN), was applied as a preprocessing procedure followed by the logistic regression analysis. The performance was evaluated by accuracy, the area under the curve (AUC), confusion matrix, and *F*_3_ score.

**Results:**

The C statistics for the Cox proportional hazards model was 0.807, while the AUC for the logistic regression analysis was 0.700, both on a comparable level to previous studies. With the model trained on the resampled set, 86.3% of patients with ESKD were correctly identified, although it was at the cost of the high misclassification rate of negative cases. The *F*_3_ score was 0.245, much higher than 0.043 for the logistic regression analysis and 0.022 for the Cox proportional hazards model.

**Conclusion:**

This study pointed out the imbalanced data structure and its effects on prediction accuracy, which were not thoroughly discussed in previous studies. We were able to identify patients with high risk for ESKD better from a clinical perspective by using the resampling method. But, it has the limitation of the high misclassification of negative cases. The technique can be widely used in other clinical topics when imbalanced data structure should be considered.

## Introduction

Chronic kidney disease (CKD) is a common chronic disease with a high prevalence ranging from 5 to 25%, according to the survey studies in European countries (1). Of all the patients with CKD, only a small proportion will progress to end-stage kidney disease (ESKD) when dialysis or kidney transplants are necessary (2, 3). ESKD is a real burden for the patient with the high mortality rate and the high cost of renal replacement therapy (4). The evolution from the early stage of CKD to ESKD takes several years in most cases (5). The guidelines of CKD based on sufficient evidence show that good follow-up treatments of CKD can slow down the progression toward ESKD (6). Therefore, an accurate predictive method that can identify patients with CKD with a high risk of ESKD at an earlier stage is essential to slow down the progression. A systematic review on prognostic models for CKD shows that previous studies mostly applied the Cox proportional hazards model (87%) or the logistic regression analysis (3%) (7) and this is also true for articles on prognostic models for ESKD (8, 9).

Despite the low prevalence or incidence of many chronic diseases, for instance in our use case, only a small portion of patients with CKD will progress to ESKD; the impact of this low incidence on the validity of prognostic models has not been thoroughly discussed in previous studies. The low incidence, e.g., 15% developing ESKD vs. 85% not developing ESKD, raises the necessity of discussion about imbalanced data structure. Imbalanced data is a dataset with a skewed distribution. Classes with a large proportion (in our case, people with CKD not developing ESKD) are called majority, while classes with a small proportion (those developing ESKD) are called minority. The degree of imbalance is regarded as moderate if the proportion of minority class is within the range of 1–20% (10). The prevalence or incidence of most chronic diseases is within this range; therefore, the imbalanced data structure is a data issue that requires attention for clinical studies.

The biggest problem caused by imbalanced data structure is called the “accuracy paradox,” which describes a situation where accuracy is unable to reflect the real performance of a model (11, 12). A model will tend to predict every case as category A if the incidence of category A is dominant, e.g., 99%, leading to perfect accuracy of 99%. In the context of clinical studies, this means the model predicts every participant as “no risk of developing a disease in the future” to achieve good performance. Not only accuracy, but also some other commonly used evaluation measures, such as precision, recall, the area under the curve (AUC), and C statistics for survival models, are not adequate performance metrics for imbalanced datasets (13).

When the model mistakenly predicts the patients as “no risk,” it is called false negative (FN). Oppositely, it is called false positive (FP) if a healthy person is predicted as “high risk to develop the disease in the future.” In the example of 99% healthy people vs. 1% patients, if the model predicts all the cases as negative, it means a 99% true negative (TN) rate and a 1% FN rate, no true positive (TP) cases, or FP cases. This is an extreme example, but most survival models or the logistic regression function in a similar way, leading to particularly high accuracy and extremely low TP rate and FP rate. However, the goal of the prognostic models is to correctly detect the TP cases; in our use case, it is to correctly predict the patients with a high risk of developing ESKD in the future. Therefore, it is necessary to provide a feasible solution that can deal with imbalanced data structure, improve the ability of the model to identify minority cases, and evaluate the performance correctly.

This study aims to introduce the use of resampling methods as data preprocessing to improve the predictive accuracy of high-risk patients for evolution toward ESKD and suggest an evaluation measure with better clinical relevance. But, it should be aware that when the model tries to improve the TP rate, the FP rate will inevitably be increased at the same time. Considering the high rate of negative cases, it will also result in a marked decrease in accuracy. It is a trade-off that should be aware of and it will be elaborated in the Discussion.

## Materials and Methods

### Patients

The study used Intego, a longitudinal database collecting electronic medical records of patients in general practice in Flanders, Belgium since 1994. Data are yearly collected from the daily consultation, including every patient who had contact with their general practitioner (GP) in that year. About 300,000 individual patients are recorded in the database, corresponding to over 2.3% of the Flemish population and this Intego population is representative of the general population in Flanders, Belgium (14). Intego contains all the coded data registered in general practices, including clinical parameters, laboratory tests, disease diagnosis, and prescriptions. The diagnosis is coded based on the International Classification of Primary Care-2 (ICPC-2) and prescriptions are based on the Anatomical Therapeutic Chemical (ATC) classification system. Intego uses an opt-out methodology and is approved by the local ethics committee of the KU Leuven and in line with Belgian and European privacy regulations.

The target population for this study is patients with CKD older than 50 years with records from 2005 to 2015. A total of 11,645 patients were involved in this study, including 11,424 patients with CKD and 221 patients who developed ESKD. Among all the patients, the average interval between the last observation and its former records was 1.358 years, meaning that this study could be broadly regarded as a 1.5-year predictive model.

### End-Stage Kidney Disease and Risk Factors

To define patients with CKD and patients with ESKD, we used the estimated glomerular filtration rate (eGFR) as recommended (15).


$$\text{eGFR}=\{\begin{array}{c} 175\;*\;\text{creatinin}{\text{e}}^{-1.154}\;*\;\text{ag}{\text{e}}^{-0.203}\;\text{if}\;\text{male}\\ 0.742\;*\;175\;*\;\text{creatinin}{\text{e}}^{-1.154}\;*\;\text{ag}{\text{e}}^{-0.203}\;\text{if}\;\text{female} \end{array}$$

Patients with CKD were defined as patients with eGFR lower than 60 *ml*/min/1.73 *m*^2^ for 3 or more months. Patients with CKD were labeled as having ESKD if they had an eGFR once lower than 15 *ml*/min/1.73 *m*^2^ and the mean of the last two measurements was lower than 30 *ml*/min/1.73 *m*^2^ (5). Each time point would be recorded in a new variable *ESKD time*, which tracked the progression of CKD. In the follow-up prediction analysis, the endpoint could be patients having ESKD for the first time or the end of the observation.

The risk factors were mainly selected referring to previous studies (5, 16–19), including age, gender, hemoglobin, uric acid, hypercholesterolemia, type 2 diabetes mellitus, obesity, hypertension, and malignancy. Apart from these variables, which were widely used in ESKD studies, we added two types of medication such as antihypertensive medication and cholesterol-lowering medication. Previous studies show that the decline in blood pressure is a strong risk factor in the decline of kidney function (19, 20) and the use of antihypertensive medications may have accelerated kidney damage (21). Besides, cholesterol-lowering medication decreases the overall risk of cardiovascular disease (CVD) and mortality of patients with CKD (22). Hence, we included these medications in this study. Some risk factors considered in other studies were not included due to a lack of sufficient information in the database, for example, tobacco use. Hemoglobin (g/dl), uric acid (mg/dl), and age are three numeric variables and all other variables for disease diagnosis or medication prescriptions are dummy variables to indicate the occurrence. A detailed description of risk factors can be found in Supplementary Table 1.

Proteinuria and eGFR at the time of diagnosis of CKD are 2 factors that are among the highest weighted risk factors for progression to ESKD. However, as mentioned above, the diagnosis of CKD and ESKD in this study was made based on eGFR. In this case, we can no longer use eGFR as the predictor. Besides, we did not have sufficient data to construct a variable to represent proteinuria. The Intego was collected in general practice. Most of the variables were from blood samples, whereas we had very limited laboratory tests collected from urine samples. The reason for the low numbers of proteinuria is 2-fold: (i) urinary analyses are not as often performed and (ii) the different laboratories use different coding values and reference values for proteinuria or albuminuria, which make these data hard to use. The model performance will be further improved, if more data (e.g., separate diagnosis records of CKD and ESKD, proteinuria, etc.) is available.

### Data Preprocessing–Resampling Methods

In this study, the resampling method was applied as a preprocessing step to solve the data issue of an imbalanced structure.

There are mainly 3 types of resampling methods, i.e., oversampling methods, undersampling methods, and a combination of over- and undersampling methods. Oversampling is a method to duplicate samples from the minority class. The representative methods include random oversampling, the Synthetic Minority Over-Sampling Technique (SMOTE), and the Adaptive Synthetic (ADASYN). All of these three methods were tested. Undersampling is a method to delete samples from the majority class. The representative methods include Random Undersampling, NearMiss (Parameter = 1, 2, or 3), Condensed Nearest Neighbor, Tomek Links, and Edited Nearest Neighbor (ENN). All of these methods were tested, including different parameters from 1 to 3 for NearMiss. A combination of over- and undersampling method is a hybrid method that duplicates samples in the minority and deletes samples from the majority. We tested the SMOTE-ENN and the SMOTE-Tomek. For this study, the SMOTE-ENN was chosen as it was the only one that could deal with the imbalanced structure without losing the original pattern and could yield the best performance when applied together with the predictive model.

The SMOTE-ENN is a combination of the SMOTE (23), one of the most popular oversampling methods, which generate new observations based on nearest neighbors determined by Euclidean distance in feature space and ENN, an undersampling method, which can improve the classification accuracy of minority instances by removing the observations from the majority class that is close to the borderline of different classes calculated by the nearest neighbor algorithm.

One characteristic of the SMOTE-ENN is that the resampled data does not have the same numbers in different classes. Instead, the resampled majority class is still larger than the minorities, but the gap in size between different classes is much closer. For example, a variable may have 100 values as *CategoryA* and 900 values as *CategoryB*. After applying the SMOTE-ENN, the resampled data may have 480 values as *CategoryA* and 670 values as *CategoryB*. Other resampling methods tend to have equal numbers in different categories, i.e., both the categories have 900 values after applying oversampling method or both have 100 values after applying the undersampling method. This preprocessing procedure was done in Python 3.6, imblearn 0.5.0.

### Statistical Analysis

We first compared the baseline characteristics of patients with CKD and ESKD. The mean and SD of continuous variables and the percentage of binary variables were calculated. The chi-squared test was conducted to check whether these variables were statistically different for patients with CKD and ESKD.

The whole data were randomly split into the train set and the test set at the ratio of 0.75:0.25 based on the patient ID. All the records in the train set were used to learn the characteristics of patients with ESKD, while in the test set, the records in the last observation year for patients with non-ESKD and the records with ESKD diagnosis for patients with ESKD were removed to create a scenario where the future status was kept unknown.

The logistic regression analysis and the Cox proportional hazards model were applied as reference models. The odds ratio (OR) for the logistic regression analysis and hazard ratio (HR) for the Cox proportional hazards model were calculated, respectively, and 95% CI was given to show the significance level of the estimated parameters.

For the Cox proportional hazards model, time-to-event outcomes were censored for loss to follow-up or end of the study. For the logistic regression analysis, the outcome was an indicator of whether to progress to ESKD during the observation period and it was defined based on the whole history of the patient in the database. The patients could have multiple records at different periods and all the records were kept without selection. Records from patients with no ESKD occurrence would be labeled as “0.” For a given patient with at least one ESKD occurrence, “1” was assigned to all the records, although some records were at the early stage when he had not developed ESKD yet. Therefore, the outcome was not to represent the temporal status of the patients; instead, it classified the participants into the two groups such as patients who would not have ESKD and patients who would have ESKD. The calculation of the logistic regression analysis and the Cox proportional hazards model was done in R software version 3.5.1.

In the next step, the SMOTE-ENN was applied on the train set for the outcome to generate resampled data including fewer observations in the majority class and more in the minority class, aiming to improve the prediction performance in the case of unbalanced data structure. Then, the logistic regression analysis was applied again on the resampled data and the performance was evaluated on the original test set and compared with the logistic regression analysis without resampling methods and the Cox proportional hazards model.

Accuracy and the AUC were the main measures to assess the prediction performance in previous studies and the C statistics was used for survival models. A confusion matrix is a table to describe the performance of the classification problem by dividing the results into four groups, namely, TP, TN, FP, and FN. As the aim of this study was to detect positive cases among all the participants, it was more important to avoid labeling patients of high risk as negative cases than monitoring a low-risk patient for a period. In this case, we used another measure, **F**_**β**_, which was a weighted harmonic mean of precision and recall. The **F**_**β**_ score is a series of scores constructed following a formula, where the parameter **β** represents the level of the penalty of FN. When the parameter is 1, FN and FP are treated equally. The score lends more weight to precision when **β** is lower than 1, whereas it favors recall when **β** is higher than 1. In this article, to alleviate the negative effect of the imbalanced data structure and take into account the clinical practice, we would like to overemphasize the recall, meaning that we give higher costs to FN than FP. As a result, we need parameters higher than 1. We compared the results by using parameters 1, 2, and 3 and decided to use 3 in this study. The results are shown and discussed in section Performance Evaluation and section Evaluation Measures.


$${\text{F}}_{\beta }=\frac{\left(1+\beta^{2}\right)\;*\;\text{Precision}\;*\;\text{Recall}}{(\beta^{2}\;*\;\text{Precision})\;+\;\text{Recall}}$$

The estimated outcome of the logistic regression analysis was the probability of developing ESKD, while that of the Cox proportional hazards model was survival probability. The default thresholds to define positive and negative cases for both the Cox proportional hazards model and the logistic regression analysis were 0.5. To examine the effects of threshold choice on the predictive performance, the confusion matrices and other evaluation measures of the logistic regression analysis (threshold = 0.7) and the Cox proportional hazards model (threshold = 0.05 and 0.01) were calculated.

## Results

### Study Participants

A total of 11,645 patients were involved in this study, including 11,424 patients with CKD and 221 patients with ESKD. Table 1 summarizes the detailed baseline characteristics of patients with CKD and ESKD. The incidence of ESKD among all the study participants was 1.90%. Based on the statistical test, *agents acting on the renin-angiotensin system, beta-blocking agents, age*, and *type 2 diabetes mellitus* were not statistically significantly different for patients with CKD and ESKD. The collinearity between all the predictors was checked by using the correlation matrix. The highest absolute values of the correlation were around 0.2; therefore, there was no severe collinearity in the data. The heatmap of the correlation matrix is added in the Supplementary Files.

**Table 1:** Baseline characteristics of patients with CKD over 50-years old in Intego (2005–2015) by future progression or no progression to ESKD.

	**CKD**	**ESKD**	***p*-value**
No. of observation (No. of patients)	58,529 (11,424)	2,257 (221)	
Hemoglobin (g/dL) (mean ± SD)	13.2 ± 1.68	11.9 ± 1.60	<0.001
Uric Acid (mg/dL) (mean ± SD)	6.5 ± 1.81	7.2 ± 2.18	<0.001
Age (year) (mean ± SD)	75.3 ± 9.66	75.5 ± 9.59	0.46
Type 2 diabetes mellitus (%)	14.3	13.0	0.09
Hypercholesterolaemia (%)	10.4	6.0	<0.001
Use of antihypertensives drugs (%)	3.9	14.9	<0.001
Use of diuretics (%)	30.1	35.7	<0.001
Use of beta blocking agents (%)	41.6	41.4	0.88
Use of calcium channel blockers (%)	17.9	31.7	<0.001
Use of agents acting on the renin-angiotensin system (%)	41.8	41.9	0.98
Use of lipid modifying agents (%)	42.4	38.0	<0.001
Obesity (%)	1.4	0.6	0.001
Hypertension (%)	15.2	11.9	<0.001
Malignancy (%)	17.3	24.0	<0.001
Male gender (%)	60.0	53.2	<0.001

*CKD, chronic kidney disease; ESKD, end-stage kidney disease*.

### Resampling Methods

The train set and the test set were divided based on the patient ID at the ratio of 0.75:0.25, so the number of patients in the train set was 75% of the total participants, but the number of observations was not strictly 75% of the total observation (44,006 CKD records and 1,631 ESKD records). After the resampling procedure, the number of CKD observations slightly decreased, while that of ESKD records grew to a large number as high as more than 26.5 times the original number (39,110 CKD records and 43,232 ESKD records). The resampling methods only changed the number of measurements, but the number of patients in the train set and the test set remained unchanged.

### Estimates of Three Models

The estimated parameters of the three models are shown in Table 2. All the estimates of the coefficients were statistically significant and *antihypertensives, calcium channel blockers, malignancy*, and *uric acid* had ORs higher than 1, meaning that they had positive effects on the probability of developing ESKD, while ORs lower than 1 indicated a negative relationship.

**Table 2:** Parameter estimates of the logistic regression analysis and the Cox proportional hazards model to predict the risk of future progression to ESKD.

	**SMOTE-ENN + Logistic regression**		**Logistic regression**		**Cox model**	
	**OR**	**95% CI**	**OR**	**95% CI**	**HR**	**95% CI**
Hemoglobin (g/dL)	0.501	0.495–0.507	0.587	0.561–0.616	0.640	0.612–0.670
Uric acid (mg/dL)	1.189	1.178–1.199	1.088	1.040–1.137	1.055	1.010–1.102
Age (year)	0.978	0.976–0.979	0.976	0.967–0.986	0.967	0.958–0.976
Type 2 diabetes mellitus	0.777	0.740–0.817	0.849	0.637–1.114	0.711	0.541–0.933
Hypercholesterolaemia	0.624	0.585–0.666	0.661	0.431–0.971	0.590	0.396–0.877
Male gender	0.550	0.531–0.569	1.341	1.110–1.619	1.211	1.007–1.456
Antihypertensives	3.095	2.884–3.323	2.684	1.963–3.618	2.697	2.005–3.628
Diuretics	0.862	0.830–0.897	1.121	0.907–1.381	1.268	1.034–1.555
Beta blocking agents	0.962	0.927–0.998	0.890	0.719–1.101	0.847	0.687–1.045
Calcium channel blockers	1.844	1.768–1.924	1.990	1.581–2.493	1.935	1.551–2.415
Agents acting on the renin-angiotensin system	0.885	0.853–0.918	0.429	0.341–0.538	0.443	0.355–0.553
Lipid modifying agents	0.761	0.733–0.791	0.865	0.693–1.077	0.722	0.581–0.897
Obesity	0.142	0.103–0.192	0.215	0.012–0.969	0.150	0.021–1.070
Hypertension	0.668	0.636–0.702	0.749	0.548–1.003	0.598	0.444–0.805
Malignancy	1.385	1.330–1.442	1.015	0.803–1.272	0.875	0.699–1.096

*OR, odds ratio; HR, hazard ratio*.

*The participants are patients with chronic kidney disease (CKD) older than 50 years old with at least 2 records from 2005 to 2015. The start of the follow-up is the first observation after 2005 and the end of follow-up is the last observation*.

The results of the logistic regression analysis of resampled data were different from the results of the logistic regression analysis and the Cox proportional hazards model of the original train set. There were two more variables, i.e., *gender (male)* and *diuretics*, indicating positive effects in the latter two models. It was also possible that the values of ORs or HRs differed even though they all meant positive or negative relations. For example, the value of estimates of *hemoglobin* gradually increased from 0.501 for the SMOTE-ENN + logistic regression analysis, to 0.587 for the logistic regression analysis, and 0.640 for the Cox proportional hazards model. A similar increasing or decreasing trend among these three models could be observed in uric acid, diuretics, beta-blocking agents, and malignancy. Besides, the estimate of ORs in the SMOTE-ENN + logistic regression analysis could have distinct values when the other two models had relatively the same values. For instance, *gender (male), antihypertensives, diuretics*, and *agents acting on the renin-angiotensin system*. Although the values of *agents acting on the renin-angiotensin system* in three models were all lower than 1, the estimated value of the SMOTE-ENN + logistic regression analysis was almost 2-fold of the other two values. In some cases, the logistic regression analysis had the highest values among the three models such as *diabetes, hypercholesterolemia, calcium channel blockers, lipid-modifying agents, obesity*, and *hypertension*; considering most of these variables were not significant in the logistic regression analysis, we could interpret this type of difference as limitation of the logistic regression analysis that it could not correctly estimate the results.

It can be found that the estimates of the SMOTE-ENN + the logistic regression analysis were very different from the other two models, which was further supported by the fact in the performance evaluation section. This was explained in the Discussion.

### Performance Evaluation

The performance of all the three models was evaluated on the original test set. There were 51 patients with ESKD and 2,858 patients with CKD in the test set. We made a prediction for the patients about their possibility to develop ESKD in the future state; therefore, there was one result for one patient as an overall prediction.

Table 3 shows the confusion matrices of three models and the threshold to decide whether the patients would develop ESKD or not were 0.5. Although the accuracy of the final results of the logistic regression analysis and the Cox proportional hazards model could be very high, the models failed to detect real patients with ESKD among all the participants. Almost all the patients were predicted as patients with non-ESKD, leading to accuracy as high as 0.981 due to the highly unbalanced data structure. In contrast, the SMOTE-ENN + logistic regression analysis detected 86.3% of patients with ESKD at the cost of misclassification of 45.2% patients with CKD.

**Table 3:** Prediction performance of the logistic regression analysis and the Cox proportional hazards model.

	**SMOTE-ENN + Logistic regression**		**Logistic regression**		**Cox model**	
	**Condition positive**	**Condition negative**	**Condition positive**	**Condition negative**	**Condition positive**	**Condition negative**
Predicted Positive	TP = 44	FP = 1291	TP = 2	FP = 6	TP = 1	FP = 2
Predicted Negative	FN = 7	TN = 1567	FN = 49	TN = 2852	FN = 50	TN = 2856
Accuracy	0.554		0.981		0.982	
AUC	0.687		0.700		0.693	
Recall	0.863		0.039		0.020	
Precision	0.033		0.250		0.333	
F_3_	0.245		0.043		0.023	

*TP, true positive; FP, false positive; TN, true negative; FN, false negative*.

*The performance of all the three models was evaluated on the original test set*.

Other evaluation measures are also shown in Table 3. It was not surprising that the SMOTE-ENN + logistic regression analysis had the lowest accuracy, 0.554, compared to the 0.981 and 0.982 of the other two models. The AUC of all the three models was relatively the same on the same level, making it difficult to distinguish the performances. If the aim was only to detect positive cases and recall was used to evaluate the model without considering the cost of misclassification of negative cases, the performance of the SMOTE-ENN + logistic regression analysis would be incredibly good with a recall of 0.863, compared with the values of 0.039 and 0.020 of the other two models. The situation would be different, if precision was used. In this case, it was more reliable to use **F**_**3**_ as the measure to evaluate the performance, as it was a combination of both the precision and recall, with a penalty on precision to address the importance of detecting patients with ESKD accurately.

### Threshold Comparison

The evaluation metrics of the results with different thresholds are shown in Table 4. The results of the Cox proportional hazards model with a threshold of 0.05 and 0.01 had a significant increase in the number of positive cases; however, the growth in positive numbers was at a higher cost of misclassification of negative cases than the SMOTE-ENN + logistic regression analysis (FP = 2,378 when threshold = 0.01). The logistic regression analysis with a threshold of 0.7 had relatively better results, accuracy = 0.752, F3 = 0.300, when compared with the results of default threshold, accuracy = 0.554, F3 = 0.245.

**Table 4:** Prediction performance of the Cox proportional hazards model with different thresholds.

	**SMOTE-ENN + Logistic regression**				**Cox model**			
	**RP = 0.7**		**RP = 0.6**		**SP = 0.05**		**SP = 0.01**	
	**Condition positive**	**Condition negative**	**Condition positive**	**Condition negative**	**Condition positive**	**Condition negative**	**Condition positive**	**Condition negative**
Predicted Positive	TP = 36	FP = 705	TP = 41	FP = 987	TP = 26	FP = 773	TP = 43	FP = 2378
Predicted Negative	FN = 15	TN = 2153	FN = 10	TN = 1871	FN = 25	TN = 2085	FN = 8	TN = 480
Accuracy	0.752		0.657		0.726		0.180	
Recall	0.706		0.804		0.510		0.843	
Precision	0.049		0.040		0.033		0.018	
F_3_	0.300		0.276		0.207		0.149	

*RP, risk probability; SP, survival probability; TP, true positive; FP, false positive; TN, true negative; FN, false negative*.

*The performance of all the three models was evaluated on the original test set*.

## Discussion

This study pointed out the imbalanced data structure and its effects on prediction accuracy, which were not thoroughly discussed in previous clinical studies. In order to correctly detect the high risk patients, we applied a resampling method called the SMOTE-ENN, which creates synthetic cases of ESKD to add to the weight of the positive outcomes and diminish the effect of the imbalanced dataset. We were able to identify patients with high risk for ESKD better than survival models and logistic regression analysis, although it was at the cost of a high misclassification rate of negative cases (45%).

### Imbalanced Data

The imbalanced data structure caused by low prevalence or incidence of some diseases was rarely discussed in previous studies. Survival models or the logistic regression analysis was applied in most studies, with the AUC or the *C* statistics as evaluation measures. As shown in Table 3, the model performance looked satisfactory only because these evaluation measures were not able to reflect the real prediction performance. The *C* statistics of the Cox proportional hazards model in this article was 0.807 and the AUC was 0.693, which were both comparable to previous studies (8, 9, 24). However, the confusion matrix showed that the good performance was caused by the fact that the models predicted almost all the results as negative cases and it could not give reasonable results by changing the survival probability thresholds.

This article proposed a solution to the imbalanced structure by applying the resampling method before the use of the logistic regression analysis. It was not difficult to introduce the resampling methods into the disease prediction, as it was more like a data preprocessing procedure, instead of adding complexity to the model. But, the simple action solved the problem of imbalanced data structure, making the logistic regression analysis able to detect the real patients with ESKD.

One of the limitations was that 45% of negative cases were misclassified, but it might be more important to accurately detect patients with ESKD than asking more patients to take good care of themselves when they were at low risk. To prevent the progression to ESKD, extra efforts include timely treatment of the underlying disease (25), antihypertensive therapy (26, 27), antidiabetic therapy (28, 29), and lifestyle management (30). In clinical practice, the medical care to prevent ESKD does not require additional medication. The identified patients with CKD with a high risk of ESKD will benefit from a more intensive follow-up examination of blood pressure, glucose, and diet, which can be done at home by themselves at low costs. Therefore, it is not a heavy physical or economic burden for patients with CKD identified as high risk.

Considering the mental burden of the diagnosis of “high risk,” patients should be explicitly informed of the misclassification rate and the true rate of progression. They need to be fully aware that the suggested risk of the model is not the definite conclusion, but a subsequent analysis needs to be followed. The data used in this study were extracted from the primary care data, which were collected without a focus on a specific disease, meaning that nephrologists can collect more data for more accurate diagnosis. Also, the average observation period was ~1.5 years, which was relatively short compared to most studies tracking ESKD. Therefore, it would be very likely that a higher proportion of patients evolve to ESKD after the observation period. This also explains the necessity of longer follow-up and closer examinations for patients with high risk. Once the high-risk patients with CKD are identified, with more frequent and may be more specific examinations, it is possible to further predict the TP cases much more accurately from all the identified patients with CKD.

Although the misclassification rate of negative cases seems to be high, significant improvements can be observed when compared with the Cox proportional hazards model. To accurately identify 44 patients with ESKD out of 51 using our method, 1,291 patients with CKD were mistakenly marked as “high risk.” However, when we used the Cox proportional hazards model (threshold = 0.01) to identify 43 patients with ESKD, 2,378 out of 2,858 patients with CKD were marked as “high risk.” The traditional prognostic models are unable to successfully detect the TP cases; it is safer to track all the patients with CKD and give them frequent examinations, which means, 1 out of 57 tracked patients will truly progress to ESKD in the future. By using our method, we narrowed the size of the tracked group and 1 out of 30 patients will be patients with ESKD in the future.

The superiority of the proposed model was also revealed when comparing the model estimates (Table 2). It was surprising that the SMOTE-ENN + logistic regression analysis had very different estimates when compared with the other two traditional models, considering the distinct classification results. Take *gender (male)* as an example, it had a negative effect on the progression of ESKD, while the other two models had positive effects. Based on the baseline characteristics in Table 1, 60% of patients with CKD were male, whereas only 53% of patients with ESKD were male. Considering all the patients were patients with CKD at the start of this study, it shows that a higher proportion of females (1 out of 45) evolved ESKD than males (1 out of 59). Therefore, the estimates of the proposed model performed better in describing the progression.

When the threshold of the logistic regression analysis was set as 0.7, the predictive performance was relatively better. About 75% of patients (70.6% patients with ESKD and 75.3% patients with CKD) were correctly predicted. The overall performance was better, but this was achieved by tolerating a lower accuracy in detecting TP cases. It could be discussed in future study to what extent the trade-off should be made, but at least it was necessary to keep in mind that FP should be penalized and that the imbalanced data structure should be specifically dealt with.

### Evaluation Measures

Because of the imbalanced data and the reality that FN and FP should not be treated equally, it was very difficult to reflect the true predicted results; thus, the evaluation measures should be carefully compared and selected. Most studies used accuracy and the AUC as evaluation measures and Table 3 reveals that they could not work well in this complex situation. Similarly, recall and precision could only show one aspect of the prediction. F-score was chosen as the evaluation measure in the end, with the parameter set as 3. Even a different choice of parameter could lead to a completely different conclusion, it stands for the level of penalty to FP. As shown in Supplementary Table 2, when the parameter was 1, meaning FN and FP were treated equally, the logistic regression analysis without any special procedures turned out to be the model with the best performance, which was not true. The SMOTE-ENN + logistic regression analysis started to gain benefits when the parameter was higher, adding a penalty to FP. When it was 2, the SMOTE-ENN + logistic regression analysis was already the best model, but the gap in the F-scores was not large enough to convince the difference. In this case, we chose to use **F**_**3**_ score as the evaluation measure in this article.

### Generalization

The topic of this article is to predict patients with ESKD out of patients with CKD. However, the same problem of imbalanced data structure can be commonly observed for many chronic diseases. As mentioned in the Introduction, it is regarded as moderate-level imbalance if the proportion of minority class is within the range of 1–20%. Few chronic diseases have a prevalence or incidence higher than 20%; instead, many chronic diseases had the prevalence within the range of 1–20%. Based on the definition of rare diseases (31), the prevalence can be as low as 5 in 10,000. Therefore, the imbalanced data structure is a data issue that requires special attention for almost all the diseases when giving prognostic predictions. The resampling technique presented in this study can be widely used for studies on other diseases in the future. It is not necessary to use the logistic regression analysis together with the SMOTE-ENN all the time; the logistic regression analysis can be replaced with some other prognostic models. The resampling technique can be regarded as a data preprocessing procedure that alleviates the issue of imbalanced data structure and can be easily combined with other methods. It is the same for the evaluation measure. F-score can be widely used when the FN and FP cases should not be treated equally.

## Data Availability Statement

The datasets presented in this article are not readily available because the data that support the findings of this study are available from Intego but restrictions apply to the availability of these data, which were used under license for the current study, and so are not publicly available. Requests to access the datasets should be directed to gijs.vanpottelbergh@kuleuven.be.

## Ethics Statement

The studies involving human participants were reviewed and approved by the Local Ethical Committee of the KU Leuven. Written informed consent for participation was not required for this study in accordance with the national legislation and the institutional requirements.

## Author Contributions

XS and GV developed the study concept and design. GV contributed to data collection. XS and TQ performed the data analysis and interpreted the results. XS drafted the manuscript. All authors provided critical revisions and approved the final version of the manuscript for submission.

## Funding

This study was supported by the KU Leuven: Research Fund (projects C16/15/059, C3/19/053, C32/16/013, and C24/18/022), Industrial Research Fund (Fellowship 13-0260), and several Leuven Research and Development bilateral industrial projects and the Flemish Government Agencies: FWO [EOS Project no 30468160 (SeLMA), SBO project S005319N, Infrastructure project I013218N, TBM Project T001919N, and PhD Grants (SB/1SA1319N, SB/1S93918, and SB/151622)], this research received funding from the Flemish Government (AI Research Program). BD and XS are affiliated to the Leuven. AI-KU Leuven Institute for AI, B-3000, Leuven, Belgium. VLAIO [City of Things (COT.2018.018), PhD grants: Baekeland (HBC.20192204), and Innovation mandate (HBC.2019.2209), Industrial Projects (HBC.2018.0405)]; the European Commission: This project has received funding from the European Research Council (ERC) under the European Union's Horizon 2020 research and innovation program (grant agreement No. 885682); (EU H2020-SC1-2016-2017 Grant Agreement No. 727721: MIDAS), KOTK foundation.

## Conflict of Interest

The authors declare that the research was conducted in the absence of any commercial or financial relationships that could be construed as a potential conflict of interest.

## Publisher's Note

All claims expressed in this article are solely those of the authors and do not necessarily represent those of their affiliated organizations, or those of the publisher, the editors and the reviewers. Any product that may be evaluated in this article, or claim that may be made by its manufacturer, is not guaranteed or endorsed by the publisher.
