# Supplementary material for: A Resampling Method to Improve the Prognostic Model of End-Stage Kidney Disease: A Better Strategy for Imbalanced Data
Source: Front Med (Lausanne). 2022 Mar 7;9:730748. doi: 10.3389/fmed.2022.730748 (PMC8935060; doi:10.3389/fmed.2022.730748)
Supplement: Supplementary file 1 [file Data_Sheet_1.PDF]

Supplementary Material

1 Supplementary Tables

Supplementary Table 1. Full list of risk factors

Risk Factors	Description / Details
Age	/
Gender	/
Hemoglobin	/
Uric acid	/
**Disease**	**ICPC Codes**
Hypercholesterolaemia	T93
Type 2 diabetes mellitus	T90
Obesity	T82, T83
Hypertension	K85, K86, K87
Malignancy	Malignancy NOS (A79), Hodgkin's disease/lymphoma (B72), Leukaemia (B73), Malignant neoplasm blood other (B74), Benign/unspecified neoplasm blood (B75), Malignant neoplasm stomach (D74), Malignant neoplasm colon/rectum (D75), Malignant neoplasm pancreas (D76), Malignant neoplasm digest other/NOS (D77), Neoplasm digest benign/uncertain (D78), Neoplasm of eye/adnexa (F74), Neoplasm of ear (H75), Neoplasm cardiovascular (K72), Malignant neoplasm musculoskeletal (L71), Neoplasm benign/unspecified musculo. (L97), Malignant neoplasm nervous system (N74), Neoplasm nervous system unspecified (N76), Malignant neoplasm bronchus/lung (R84), Malignant neoplasm respiratory, other (R85), Neoplasm respiratory unspecified (R92), Malignant neoplasm of skin (S77), Malignant neoplasm thyroid (T71), Neoplasm endocrine other/ unspecified (T73), Malignant neoplasm of kidney (U75), Malignant neoplasm of bladder (U76), Malignant neoplasm urinary other (U77), Neoplasm urinary tract NOS (U79), Malignant neoplasm relate to pregnancy (W72), Malignant neoplasm cervix (X75), Malignant neoplasm breast female (X76), Malignant neoplasm genital other (f) (X77), Genital neoplasm other/unspecified (f) (X81), Malignant neoplasm prostate (Y77), Malignant neoplasm male genital other (Y78) and Benign/unspecified neoplasm gen. (m) (Y79)
**Medication**	**ATC Codes**
Antihypertensives	C02
Diuretics	C03
Beta blocking agents	C07
Calcium channel blockers	C08
Agents acting on the renin–angiotensin system	C09
Lipid modifying agents	C19

Supplementary Table 2. Comparison of F scores with different parameters to show the effect of the choices of evaluation measures

	**F1**	**F2**	**F3**
SMOTE-ENN+Logistic Regression (RP=0.5)	0.064	0.143	0.245
SMOTE-ENN+Logistic Regression (RP=0.6)	0.076	0.166	0.276
SMOTE-ENN+Logistic Regression (RP=0.7)	0.091	0.190	0.300
Logistic Regression	0.068	0.047	0.043
Cox Model (SP=0.5)	0.037	0.024	0.022
Cox Model (SP=0.05)	0.061	0.130	0.082
Cox Model (SP=0.01)	0.035	0.207	0.149

Note: RP = Risk Probability threshold, SP = Survival Probability threshold

2 Supplementary Figures

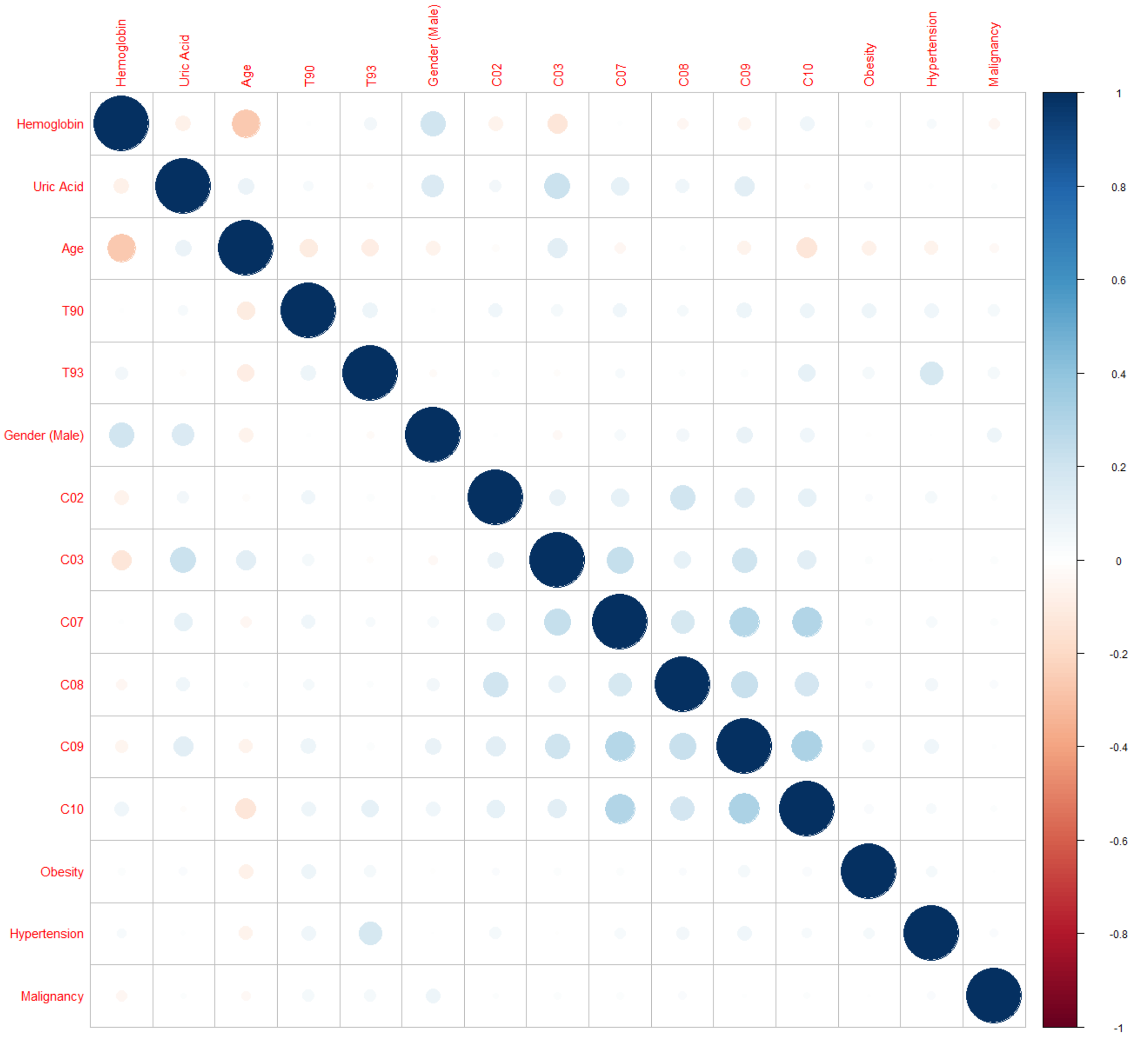

Supplementary Figure 1. The heatmap of the correlation matrix of all predictors. (Note: T90 - Type 2 Diabetes Mellitus, T93 – Hypercholesterolaemia, C02 – Antihypertensives, C03 – Diuretics, C07 - Beta blocking agents, C08 - Calcium channel blockers, C09 - Agents acting on the renin-angiotensin system, C10 – Lipid Modifying Agents.)
